# Plasma Levels of ADAMTS-13 Antigen, ADAMTS-13 Inhibitor, ADAMTS-13 Activity, and Von Willebrand Factor in Patients with Behçet’s Disease

**DOI:** 10.31138/mjr.020524.pla

**Published:** 2024-11-07

**Authors:** Uğur Karasu, Merve Erkek, Firdevs Ulutaş, Aydın Demiray, Yeşim Çimen, Serdar Kaymaz, İbrahim Açıkbaş, Hande Şenol, Veli Çobankara

**Affiliations:** 1 Division of Rheumatology;; 2 Department of Internal Medicine;; 3 Department of Medical Genetics;; 4 Department of Family Medicine;; 5 Department of Medical Biology;; 6 Department of Medical Biostatistics, Pamukkale University Faculty of Medicine, Pamukkale, Turkey

**Keywords:** Behçet’s disease, ADAMTS-13, VWF

## Abstract

**Background:**

Behçet’s Disease (BD) is characterised by recurrent aphthous oral and genital ulcers. Vascular involvement is one of the poor prognostic factors. Previously, von Willebrand Factor (VWF) has been detected higher in BD compared with healthy controls. We hypothesised that decreased activity or increased inhibitor levels of ADAMTS-13 may cause increased levels of VWF. Therefore, we investigated ADAMTS-13 in patients with BD.

**Methods:**

We included in total of 42 patients with BD and 41 healthy controls (HCs) in this cross-sectional study. Parametric data with normal distribution were compared with Student’s t-test and ANOVA, and nonparametric data with non-normal distribution were compared with Mann Whitney U and Kruskal Wallis tests.

**Results:**

The patients showed lower ADAMTS-13 antigen, lower ADAMTS-13 inhibitor, lower ADAMTS-13 activity, and higher VWF levels compared with HCs. ADAMTS-13 activity was higher in vascular involvement compared with non-vascular involvement (18.26 ± 7.3 vs 12.05 ± 6.49, p=0.012). VWF levels were also similar between vascular and non-vascular subgroups.

**Conclusion:**

Reduced ADAMTS-13 activity and increased VWF levels were detected in BD. This change has not been seen in vascular BD. The underlying pathogenetic mechanisms seem more complex in the formation of thrombosis.

## INTRODUCTION

Behçet’s Disease (BD) is a well-known multisystemic vasculitis with recurrent aphthous oral and genital ulcers. Involvement of the vascular, ocular, and/or central nervous system can present with severe life-threatening complications.^1^

Von Willebrand Factor (VWF) is an ultra-large glycoprotein that plays an important role as an endothelial cell product in plug formation via mediating platelet aggregation and adhesion to injured endothelium. VWF also stabilises Factor VIII by protecting it from proteolytic inactivation and, as a result, improves the coagulation system.^2^ Beyan E et al. showed higher VWF antigen levels in BD compared with healthy controls (HCs). Serum VWF antigen levels were correlated with ferritin levels. However, there was no relationship between plasma VWF antigen levels and erythrocyte sedimentation rate (ESR), C-reactive protein (CRP).^3^ In addition, Demirer et al, detected higher VWF antigen levels in vascular Behçet’s patients compared with patients without vascular involvement.^4^ Higher VWF antigen levels were related to poor prognosis with larger vascular involvement. ^5^ In BD, anti-endothelial cell antibodies were higher in active disease related to vasculitic changes.^6^ However, mean VWF antigen levels were detected higher in BD compared with controls, without any correlation with increased anti-endothelial cell antibodies. This condition led to research on other factors that increase VWF antigen levels except endothelial damage.^7^

Vascular involvement is one of the most important clinical manifestations. It is commonly seen in male patients with poor prognosis. The venous disease presents with recurrent attacks of venous thrombosis while arterial disease presents with the formation of aneurysms in the aorta, pulmonary arteries, and visceral arteries.^8^ There are many steps in the mechanism of thrombosis, one of which is the disruption of the combination between VWF and ADAMTS-13 (a disintegrin and metalloproteinase with thrombospondin type 1 motif, member 13). ADAMTS-13 is a disintegrin, also called VWF-cleaving protease that controls the size of VWF. The balance between the VWF and ADAMTS-13 is essential for a healthy coagulation system. Disruption of the normal VWF/ADAMTS-13 axis (usually increased VWF/ADAMTS-13 ratio) promotes aggrevated platelet response and consequently thrombosis. Patients with a deficiency of the VWF present with bleeding, whereas severe deficiency (<10% of normal levels) of ADAMTS-13 causes thrombotic thrombocytopenic purpura.^9^ Severe deficiency in ADAMTS-13 activity is caused by the presence of inhibitory antibodies against ADAMTS-13 or gene polymorphisms. Also, thrombotic microangiopathies, disseminated intravascular coagulopathy and chronic liver diseases are well known with decreased ADAMTS-13 levels.^10^ In immune thrombotic thrombocytopenic purpura (TTP), patients have anti-ADAMTS-13 immunoglobulin G antibodies that inhibit ADAMTS-13 activity or increase ADAMTS-13 clearance. Thus, this condition causes VWF-dependent microvascular thrombosis.^11^ In this study, we investigated the plasma levels of ADAMTS-13 antigen, ADAMTS-13 inhibitor, and ADAMTS-13 activity in patients with BD. We hypothesized that decreased activity or increased inhibitor levels of ADAMTS-13 may cause increased levels of VWF.

## MATERIALS AND METHODS

This cross-sectional study was carried out in the Department of Rheumatology of the Pamukkale University Faculty of Medicine, with the approval of the local ethical committee dated 02.08.2018 and numbered 60116787-020/51912. All patients with BD and healthy individuals collected within 6 months after receiving ethics committee approval were included in this study. All of the patients fulfilled the International Study Group criteria for diagnosis.^12^ They were between 18–65 years old, and had inactive BD. We used Behçet’s Disease Momentary Activity Form (BHAF), and Behçet Syndrome Activity Scale (BSAS) to evaluate disease activity. Patients with active disease, or additional inflammatory disease were excluded. We evaluated all demographic data and clinical and laboratory findings and treatment modalities of patients at the first clinic visit. The healthy control group was voluntarily obtained among people who visited the rheumatology outpatient clinic at the same time interval. Every patient is evaluated by comprehensive medical history and physical examination. Individuals with subsequent features were excluded: Having chronic inflammatory and/or vascular diseases, having infectious diseases in the recent three months, being on the medical treatment.

### VWF and ADAMTS-13

For plasma levels of VWF and ADAMTS-13, 8–10 cc of blood was collected between 8-10 am after a 12-hour fast from each patient. Blood samples prepared for VWF were centrifuged at 450 nm. ADAMTS-13 analysis was carried out with enzyme-linked immunosorbent assay (ELISA) (Technozym ADAMTS-13 Antigen, Technozym ADAMTS-13 Activity, Technozym ADAMTS-13 Inhibitor, DiaPharma Group, Inc. West Chester, OH 45069, USA). The normal reference range for ADAMTS-13 activity is between 40–130%. The normal reference range for ADAMTS-13 antigen is 0.41–1.41 IU/mL whereas ADAMTS-13 inhibitors are <12U/mL for negative samples, 12–15U/mL for borderline samples, >15U/mL for positive samples.^13^

VWF analysis was carried out using Elabscience test kits produced by the ELISA method (Elabscience ELISA Kits, Bionovation Inc. USA). The necessary steps for the reactions were prepared by the protocol determined by commercial companies. This ELISA kit recognised human VWF concentrations in the detection range of 1.56–100 ng/mL (sensitivity 0.94 ng/mL) without any coross-reactivity or interference between human VWF and analogues.

### ADAMTS-13 Gene Polymorphisms

ADAMTS-13 gene profile was performed by collecting 2 ml of blood in ethylenediaminetetraacetic acid (EDTA) tubes and using the purification kits (Applied Biological Materials Inc, Cat. No. 11352051, Canada). All samples were stored at −80 degrees to protect DNA material long term. Then, the relevant probe for ADAMTS-13 gene polymorphism was analysed with the Applied Biosystem 5000 real-time device.

### Statistical Analysis

In our study, SPSS (Statistical Package for the Social Sciences) version 22.0 (IBM, Armonk, NY, USA) was used for statistical analysis of the data. Descriptive statistics were expressed as mean ± standard deviation or median (minimum-maximum) for discrete and continuous numerical variables and as number of cases and (%) for categorical variables. Cross-tabulation statistics were used to compare categorical variables (Pearson Chi-square, Fisher). Parametric data with normal distribution were compared with Student’s t-test and ANOVA, and nonparametric data with non-normal distribution were compared with Mann Whitney U and Kruskal Wallis tests. Spearman and Pearson correlation tests were used to compare numerical variables. Results were defined as p<0.05 statistical significance.

## RESULTS

In total, 42 patients with BD and 41 HCs were included in this study. There was no statistical difference in terms of body mass index and age between the both groups. The majority of patients were male (n=25) in the Behçet group with a statistical significance (p=0.001).

**Figure 1.**
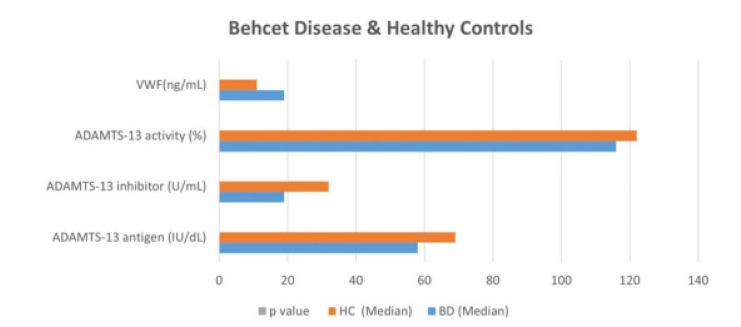
Plasma levels of ADAMTS-13 antigen, ADAMTS-13 inhibitor, ADAMTS-13 activity, and VWF in whole study group. The median values of these parameters were as follows subsequently in Behçet patients: ADAMTS-13 antigen: 58 IU/dL, p<0.05; ADAMTS-13 inhibitor: 19 U/mL, p<0.05; ADAMTS-13 activity: 116%, p<0.05; VWF: 19 ng/mL, p<0.05.

All of the Behçet patients had mucocutaneous manifestations. Seven (7) patients had vascular involvement (deep venous thrombosis in all patients), 21 patients had eye involvement, and others (14) had isolated mucocutaneous, central nervous system, and gastrointestinal system involvement. The mean time of disease duration is 97.40 ± 68.99 months in BD. 21 patients with BD were on TNF-alpha treatment, others were on different treatment modalities including azathioprine, colchicine, mycophenolate mofetil, and systemic glucocorticoids. The patients showed lower ADAMTS-13 antigen, lower ADAMTS-13 inhibitor, lower ADAMTS-13 activity, and higher VWF levels compared with HCs (**Figure 1**).

All seven patients with vascular BD had deep venous thrombosis with mucocutaneous findings. These parameters were also compared in patients with vascular and non-vascular BD. Non-vascular subgroup included all remaining patients without vascular symptoms and/or complications. ADAMTS-13 activity was higher in vascular involvement compared with non-vascular involvement (18.26 ± 7.3 vs 12.05 ± 6.49, p= 0,012). VWF levels were similar between vascular and non-vascular subgroups (**Table 2**).

**Table 1.** Demographic data of the study group.

**Variables**	**BD (n=42)**	**HCs (n=41)**	**P value**
Female/Male (n)	17/25	31/10	p=0.001
Age, years (Mean ± SD)	37.73 ± 9.18	35.48 ± 9.08	p=0.264
BMI	25.46 ± 4.21	25.39 ± 4.50	p=0.212

**Table 2.** Plasma levels of ADAMTS-13 antigen, ADAMTS-13 inhibitor, ADAMTS-13 activity, and VWF in vascular and non-vascular Behçet patients.

**Variables**		**ADAMTS-13 antigen (IU/mL)**	**ADAMTS-13 inhibitor (U/ml)**	**ADAMTS-13 activity (%)**	**VWF (ng/ml)**
**Vascular BD (n=7)**	**Mean ± SD**	0.57 ± 0.11	18.96 ± 15.6	18.26 ± 7.3	18.81 ± 4.49
**Non-vascular BD (n=35)**	**Mean ± SD**	0.64 ± 0.34	18.87 ± 16.11	12.05 ± 6.49	17.89 ± 8.98
**p value**		0.174^*^	0.729^*^	0.012^*^	0.99^*^

P value < 0,05 statistically significant.

* Mann Whitney U Test. BD: Behçet’s Disease; VWF: Von Willebrand Factor.

Single nucleotide variants including rs685523 and rs11575933 which are related to ADAMTS-13 were also examined. Heterozygous/mutant rs11575933 polymorphism was more usual in BD, whereas wild-type rs11575933 polymorphism was more common in HCs with a statistical significance (p=0.001). However, rs685523 polymorphisms were similar between BD and HCs (**Table 3**).

**Table 3.** Distribution of the rs685523 and the rs11575933 gene polymorphyms in the whole study group.

	**rs685523**		**rs11575933**	
	**Wild**	**Heterozygous/mutant**	**Wild**	**Heterozygous/mutant**
**BD**	26	16	26	16
**HC**	20	21	40	1
**p value**	0.326^*^		0.001^*^	

* Pearson Ki-Kare test.

BD: Behçet’s disease; HCs: healthy controls.

There was no statistical difference when we examined a correlation between ADAMTS-13 antigen, ADAMTS-13 inhibitor, ADAMTS-13 activity, and the above-mentioned single nucleotide variants (**Tables 4** and **5**).

**Table 4.** The correlation of the rs685523 polymorphysms with ADAMTS-13 antigen, ADAMTS-13 inhibitor and ADAMTS-13 activity.

		**rs685523 (w)** **Mean ± SD**	**rs685523 (h+m)** **Mean ± SD**	**p value**
**ADAMTS-13 antigen (IU/mL)**	**BD**	0.53 ± 0.14	0.61 ± 0.11	0.979^*^
	**HC**	0.72 ± 0.13	0.65 ± 0.13	0.183^*^
**ADAMTS-13 inhibitor (U/ml)**	**BD**	21.02 ± 17.01	20.96 ± 13.9	0.613^*^
	**HC**	33.21 ± 19.41	34.02 ± 22.5	0.990^*^
**ADAMTS-13 activity (%)**	**BD**	113 ± 12	109 ± 13	0.476^*^
	**HC**	117 ± 15	121 ± 10	0.473^*^

* Mann Whitney U test. BD: Behçet’s disease; HCs: healthy controls.

**Table 5.** The correlation of the rs11575933 polymorphysms with ADAMTS-13 antigen, ADAMTS-13 inhibitor and ADAMTS-13 activity.

		**rs11575933 (w)** **Mean ± SD**	**rs11575933 (h+m)** **Mean ± SD**	**p value**
**ADAMTS-13 antigen (IU/mL)**	**BD**	0.621 ± 0.12	0.63 ± 0.16	0.688^*^
	**HC**	0.69 ± 0.14	0.58 ± 0.19	1.00^*^
**ADAMTS-13 inhibitor (U/ml)**	**BD**	22.4 ± 17.3	18.03 ± 12.9	0.669^*^
	**HC**	34.06 ± 20.8	35.06 ± 20.8	0.390^*^
**ADAMTS-13 activity (%)**	**BD**	114 ± 12	117 ± 14	0.836^*^
	**HC**	119 ± 13	118 ± 15	0.537^*^

* Mann Whitney U test. BD: Behçet’s disease; HCs: healthy controls.

## DISCUSSION

We conducted this study to evaluate whether decreased ADAMTS-13 activity could be related to increased VWF in BD. We found increased VWF in BD by supporting recent literature, in addition to decreased ADAMTS-13 activity. Recent literature has emphasised the relationship between the presence of ADAMTS-13 inhibitor and autoimmune process.^14^ BD is not a classical auto-immune disease, nevertheless there are a few case reports in which TTP occurs in association with BD. Jabr FI et al. have defined first TTP case without any trigger in BD.^15^ Related to cyclosporin-derived endothelial injury, TTP has also developed in a patient with BD.^16^ Besides, a 31-year-old Caucasian male patient with recurrent thrombotic thrombocytopenic purpura was later diagnosed with BD. The patient developed meningoencephalitis attributed to Neuro-Behçet syndrome with normal ADAMTS-13 activity.^17^ The clinical importance of ADAMTS13 activity in TTP was so accepted that recombinant ADAMTS13 was successfully used in a patient with hereditary TTP as a new advancement.^18^ To our knowledge, this is the first cross-sectional study that has investigated ADAMTS-13 in BD. Although VWF is related to thrombosis with clinical conflicting results in studies, VWF was similar in vascular and non-vascular BD subgroups. It is difficult to interpretate clinical importance of this result due to limited number of patients with vascular involvement. Similar to our study, ADAMTS-13 activity and ADAMTS-13 activity/VWF antigen ratio were significantly lower and VWF antigen levels were significantly higher in patients with Sickle Cell Disease when compared to the controls.^19^ Despite the difference with healthy controls, they have detected no clinical importance and association with the use of hydroxyurea in these patients.

However, it is difficult in our study to explain how plasma levels of each activity, antigen, and inhibitor of ADAMTS-13 are lower in BD compared with HCs. Many functional, genetic, environmental, and disease-related pathways affect ADAMTS-13.^20^ Also, it has shown that the determination of ADAMTS-13 inhibitors or deficiency is method-dependent.^21^ Klonizakis P et al., showed higher anti-ADAMTS-13 autoantibodies in patients with positive anti-dsDNA. Patients with active systemic lupus erythematosus (SLE) or considerable cumulative tissue damage may have reduced ADAMTS-13 levels potentially mediated by ADAMTS-13 autoantibodies.^22^ Decreased ADAMTS-13 activity in combination with increased VWF levels potentially demonstrates thrombotic risk in active SLE with aPLs.^23^

In the recent literature, VWF has been repeatedly investigated; however, there are a few conflicting results. The patients with ocular and vascular involvement had higher VWF antigen compared with the control group related to disease execerbation. ^5^ Before have been emphasised, higher levels of VWF can originate from endothelial destruction due to vasculitis in the active BD.^24^ Lee LY et al. have also evaluated the levels of VWF in BD. The plasma homocysteine concentrations in the thrombosis patients were positively correlated with plasma VWF levels; a relationship which suggests injury of the vascular endothelium.^25^ Alkaabi JK et al. declared increased VWF antigen as an acute phase reactant. High VWF antigen was not related to increased propensity for thrombosis risk while they emphasised vasculitic endothelial changes as a risk factor for thrombosis.^26^ It has been shown that the level of VWF is increased and the level of ADAMTS-13 is decreased in post-op patients related to endothelial injury. But this ratio (postoperative VWF antigen/ADAMTS-13) is not associated with perioperative thrombotic events.^27^ So, the clinical relevance of ADAMTS-13 and VWF is not clear in clinical practice to predict vascular thrombosis. Consequently, increased VWF may be related to inflammation markers without presence of thrombosis. ADAMTS-13 activity was higher in vascular BD with thrombosis than non-vascular subgroup. This condition may be attributed to small patient size in the vascular group or can be explained by other inflammatory pathways in the thrombosis of BD.

The rs685523 was also investigated in SLE patients. As a result of the study, it was not correlated with stroke and anticardiolipin antibodies.^28^ Pagliari MT et al. investigated rare ADAMTS-13 variants in patients with deep vein thrombosis. They determined many protective variants in ADAMTS-13 (rs28641026, rs28503257, rs685523^*^, rs3124768, rs3118667, rs739469, rs3124767).^29^ However, the variant rs685523 polymorphism was similar between BD and HCs in our study. Not only ADAMTS-13 variants but also VWF and FVIII variants can be responsible for plasma levels of these molecules and thrombosis risk.

## LIMITATIONS

A lack of patient subgroups (especially vascular subgroup) is one of the most important limitations. Moreover, it is a single center experience, that limits results of gene polymophism studies compared to a population-based large studies. Also, we think that the presence of patients with active disease could enhance the quality of the study.

## CONCLUSION

Increased VWF with decreased ADAMTS-13 activity was detected in BD without correlation with vascular involvement. Synchronously decreased levels of ADAMTS-13 antigen and inhibitor led us to think of other underlying possible complex mechanisms. Hopefully, further studies with more patients and the comparison of active and inactive Behçet subgroups will provide more information in this field.

## ETHICAL APPROVAL AND CONSENT

This cross-sectional study was carried out in Pamukkale University Faculty of Medicine, Department of Rheumatology with the approval of the local ethical committee dated 02.08.2018 and numbered 60116787-020/51912.

## CONSENT FOR PUBLICATION

All authors accepted final version of the text for publication.

## AVAILABILITY OF DATA AND MATERIALS

Not applicable.

## CONFLICT OF INTEREST

All authors declare that they have no conflict of interest.

## FUNDING

This study was supported by the decision of the Pamukkale University Scientific Research Projects Coordination Unit dated 20.04.2019 and numbered 2019TIPF004.

## AUTHORS’ CONTRIBUTIONS

Analysing and Collection of Data: UK; ME, FU. Statistical Analysis: SK, AD, YÇ, İA, HŞ. Writing of text: FU, ME, UK. Review of the main text: VÇ, UK.
